# The role of high mobility group box-1 on the development of diabetes complications: A plausible pharmacological target

**DOI:** 10.1177/14791641241271949

**Published:** 2024-09-13

**Authors:** Nokwanda N Ngcobo, Ntethelelo H Sibiya

**Affiliations:** 1 Discipline of Pharmaceutical Sciences, School of Health Science, 56394 University of KwaZulu-Natal, Durban, South Africa; 2 59100 Pharmacology Division, Faculty of Pharmacy, Rhodes University, Grahamstown, South Africa

**Keywords:** HMGB-1, diabetes complications, diabetic nephropathy, diabetic neuropathy, diabetic retinopathy

## Abstract

**Background:**

Diabetes mellitus has emerged as a pressing global concern, with a notable increase in recent years. Despite advancements in treatment, existing medications struggle to halt the progression of diabetes and its associated complications. Increasing evidence underscores inflammation as a significant driver in the onset of diabetes mellitus. Therefore, perspectives on new therapies must consider shifting focus from metabolic stress to inflammation. High mobility group box (HMGB-1), a nuclear protein regulating gene expression, gained attention as an endogenous danger signal capable of sparking inflammatory responses upon release into the extracellular environment in the late 1990s.

**Purpose:**

Given the parallels between inflammatory responses and type 2 diabetes (T2D) development, this review paper explores HMGB-1’s potential involvement in onset and progression of diabetes complications. Specifically, we will review and update the understanding of HMGB-1 and its inflammatory pathways in insulin resistance, diabetic nephropathy, diabetic neuropathy, and diabetic retinopathy.

**Conclusions:**

HMGB-1 and its receptors i.e. receptor for advanced glycation end-products (RAGE) and toll-like receptors (TLRs) present promising targets for antidiabetic interventions. Ongoing and future projects in this realm hold promise for innovative approaches targeting HMGB-1-mediated inflammation to ameliorate diabetes and its complications.

## Introduction

Diabetes mellitus (DM) is a metabolic disease characterised by long-lasting hyperglycaemia resulting from defects from insulin secretion, insulin action, or both. Hyperglycaemia places further stress on the β-cells and establishes a negative feedback loop through which metabolic decompensation worsens β-cell failure and insulin resistance. The response of plasma insulin to glucose does not provide information about the health of the β-cell. The β-cell only responds to an increase in plasma glucose concentration with an increase in plasma insulin, and this feedback loop is influenced by the severity of insulin resistance. Thus, β-cell function is best characterized by the insulin secretion/insulin resistance.^1^

**Figure 1.**
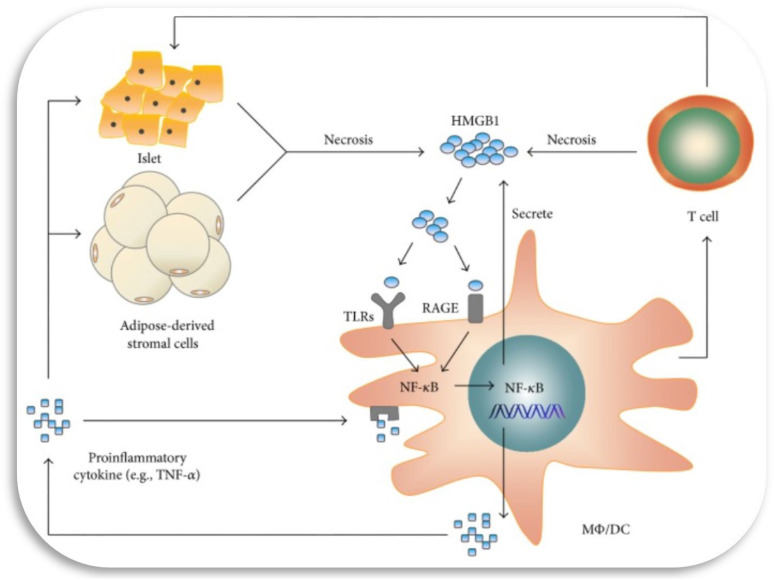
The potential involvement of HMGB-1 in type 2 diabetes is linked to early inflammation occurring in adipose tissue and pancreatic islets, leading to the necrosis of adipose-derived stromal cells and islet cells. When these cells undergo necrosis, they release HMGB-1, which then activates Toll-like receptors (TLRs) and the receptor for advanced glycation end products (RAGE) on macrophages and dendritic cells. This activation of TLRs and RAGE prompts the translocation of NF-κB into the nucleus, where it promotes the expression of inflammatory genes, including HMGB-1 itself. Furthermore, the activated macrophages and dendritic cells actively secrete HMGB-1, thereby exacerbating the necrosis of adipose tissue and pancreatic islets.

On average it takes 7 years for a person to be diagnosed with type 2 diabetes (T2D), as symptoms can be mild and may develop gradually. As a result, 30% people with T2D will already have developed complications by the time they are diagnosed. The complications include cardiovascular diseases, retinopathy, nephropathy, and neuropathy.^2^ Central to the onset and progression of diabetes complications is uncontrolled hyperglycaemia. From an aetiology perspective, these complications are associated with increases in oxidative stress, low grade inflammation and growth factors expression. Recent developments in diabetes mellitus complications pathogenesis have associated high mobility complex box (HMGB-1) with the onset and progression of diabetes complications, mainly through activation and mediating the expression of inflammatory signalling molecules (refer Figure 1).^3^ In this review, we intend to provide and consolidate recent developments on HMGB-1 and diabetes complications pathogenesis. Furthermore, we intend to explore HMGB-1 antagonism as a plausible pharmacological target for the management of diabetes complications. We envisage this review shall be more instrumental in persuading more studies towards exploring HMGB-1 suppression and antagonism in the delaying the onset and progression of diabetes complications.

## HMGB-1 box

Initially recognized for its involvement in gene expression regulation, HMGB-1, a nuclear protein, has recently been implicated in alarming pathogenic activities.^3^ This includes its role in activating proinflammatory responses upon passive release from necrotic cells or active secretion by activated immune cells into the extracellular environment.^4^ Once released into the extracellular environment, HMGB-1, partakes in various processes, including immune response, cell migration, cell differentiation, proliferation, and tissue regeneration. Notably, HMGB-1 has been associated with numerous inflammatory diseases, including cancer, trauma, arthritis,^5^ ischemia-reperfusion injury,^6^ sepsis,^7^ cardiovascular shock, diabetes, and autoimmune diseases. Furthermore, HMGB-1 has demonstrated its pivotal role as an acute inflammation coordinator across various stress models (refer Figure 1).^3,8^

HMGB-1, widely distributed across mammalian tissues and prevalent in all vertebrate nuclei, was initially identified as a nuclear protein. Within the nucleus, HMGB-1 binds to DNA and oversees several crucial DNA processes, including gene transcription, DNA replication, and DNA repair to mention a few. For a considerable time, it was exclusively acknowledged as a nuclear protein until 1999 when Wang et al (1999), first reported its involvement as a late inflammatory mediator, linking it to the pathogenesis of sepsis. Subsequently, recognition grew regarding HMGB-1’s significant role in inflammation processes.^9^ In the late 1990s, HMGB-1, initially known for its role in regulating gene expression as a non-histone nuclear protein, was rediscovered as an endogenous danger signal molecule.^4^ HMGB-1 is released from cells through two mechanisms: passively or actively.^10^ When cells are damaged or undergo necrosis, they passively release their nuclear HMGB-1, triggering an immediate inflammatory response mediated by pro-inflammatory cytokines such as tumor necrosis factor α (TNF-α).^11^ Cells lacking HMGB-1 show a delayed onset of the inflammatory response, as they are unable to activate monocytes during necrosis, as demonstrated by Scaffidi and colleagues in 2002.^11^ Additionally, HMGB-1 can be actively secreted from various cell types, including immune cells, endothelial cells, platelets, neurons, astrocytes, and cancer cells, either in response to stress or as a reinforcement to other damage-associated molecular pattern (DAMP) signals.^10^ HMGB-1’s release into the extracellular environment was found to trigger inflammatory responses. This finding coincided with the recognition of similarities in the inflammatory responses contributing to the development of T2D.^8^ Studies have shown HMGB-1’s significant role in insulin resistance and diabetes.

Under physiological conditions, HMGB-1 is present in the plasma at relatively low levels. Studies have reported normal plasma concentrations of HMGB-1 in healthy individuals ranging from 0 to 10 ng/mL.^12^ In individuals with T2D, HMGB-1 levels are significantly elevated compared to healthy controls.^13^ This elevation is associated with chronic inflammation, oxidative stress, and insulin resistance characteristic of T2D.^14^ Research indicates that plasma HMGB-1 levels in patients with T2D can be higher than 10 ng/mL, often reaching up to 20 ng/mL or more.^15^ Elevated HMGB-1 levels in these patients are correlated with poor glycaemic control and complications such as cardiovascular disease.^16^

Similarly, obesity, which is often linked to T2D, also shows increased levels of HMGB-1 in the plasma.^13^ Obesity is characterized by a state of chronic low-grade inflammation, and HMGB-1 is thought to contribute to this inflammatory milieu.^17^ Plasma HMGB-1 levels in obese individuals are often elevated, with studies showing increases comparable to those seen in T2D patients.^18,19^ The levels in obese individuals can vary but typically range between 5 and 15 depending on the severity of obesity and associated metabolic disturbances.^18^

It is worth noting that the concentration of HMGB-1 in the serum can serve as an indicator of glucose toxicity, atherosclerosis, and diminished β-cell function in individuals with diabetes.^20^ There is a positive correlation between serum HMGB-1 levels and glucose metabolism indicators such as HbA1c and fasting plasma glucose (FPG).^20^ HMGB-1 enhances the expression of ATG7 and the LC3B II/I ratio, decreases p62 expression, and promotes autophagy as studied by Zhang et al.^21^ Furthermore, serum HMGB-1 levels show a positive correlation with fasting insulin (FINS) and homeostasis model assessment-insulin resistance (HOMA-IR), and a negative correlation with HOMA-β, indicating that HMGB-1 might impair β-cell function and heighten insulin resistance.^20^ HMGB-1 contributes to insulin resistance by upregulating receptor for advanced glycation end-products (RAGE) expression, activating the TLR4/JNK/NF-κB pathway, and inhibiting the IRS-1 signaling pathway.^22,23^

## The association between diabetes and HMGB-1

Insulin resistance denotes a diminished ability of insulin to facilitate glucose uptake by the target tissues. Essentially, it signifies a condition wherein cells inadequately respond to circulating insulin.^24^ Peripheral tissues such as the liver, skeletal muscle, and adipose tissue can all serve as sites for insulin resistance.^25^ Research has shown that infiltration of macrophages into adipose tissue correlates with elevated serum insulin concentration, suggesting that macrophage-mediated inflammatory responses may significantly contribute to insulin resistance.^26^ Recent research indicates a close correlation between diabetes and HMGB-1.

**Figure 2.**
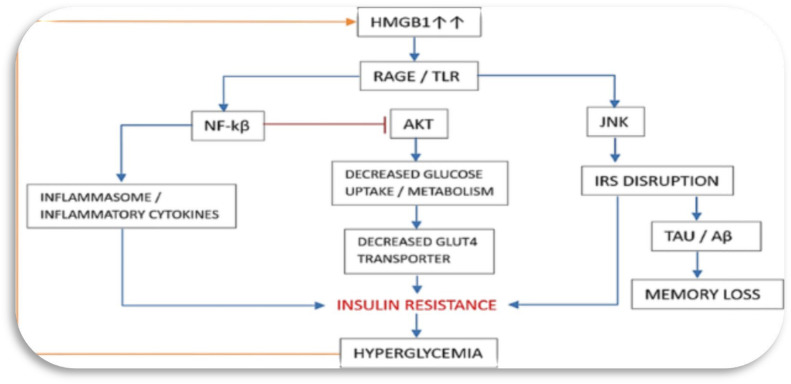
HMGB-1, a pro-inflammatory molecule, plays a significant role in the development of insulin resistance, a key factor in diabetes complications. It contributes to this condition by activating inflammatory pathways, notably NF-κB and JNK. Activation of these pathways leads to the disruption of insulin receptor substrate (IRS) function, which is crucial for the proper signalling of insulin. When IRS function is impaired, the insulin signalling pathway is compromised, resulting in decreased expression of glucose transporter 4 (GLUT4) on the cell surface. GLUT4 is essential for the uptake of glucose into cells, and its reduced expression leads to decreased glucose uptake and elevated blood glucose levels. Additionally, HMGB-1 activation increases the production of inflammatory cytokines, further exacerbating insulin resistance and inflammation. This combination of impaired insulin signalling and increased inflammation contributes to the overall metabolic dysfunction seen in diabetes and its complications, such as cardiovascular disease, neuropathy, and nephropathy.

An increase NF-κB activity has been observed in obese animal models, and blocking NF-κB has been shown to protect mice on a high-fat diet from insulin resistance.^27^ Activation of the NF-κB signalling pathway can be initiated by pattern recognition receptors like toll-like receptors (TLRs) and the RAGE, both of which interact with HMGB-1.^8^ This suggests that HMGB-1 might play a crucial role in insulin resistance through NF-κB signalling (refer Figure 2).^28^ Several human studies in obese or T2D individuals have reported elevated circulating levels of HMGB-1, which positively correlate with insulin resistance as measured by the HOMA-IR.^28^

Additionally, Dasu et al (2010),^29^ found higher circulating levels of HMGB-1 in T2D patients compared to controls, a phenomenon also supported by Škrha et al (2012).^30^ Hagiwara et al (2008), demonstrated that hyperglycaemia induced by glucose infusion in a rat model was linked to elevated serum HMGB-1 levels.^30^ Further research by Dasu and colleagues (2010), revealed elevated levels of HMGB-1 in individuals with T2D, positively correlating with TLR2 and TLR4, body mass index (BMI), and HOMA-IR.^29^ Additionally, the expression of MyD88 and NF-κB p65 was increased. The activation of TLR-MyD88-NF-κB signalling resulted in elevated cytokine levels.^28^ Subsequently, Chen et al. found significantly upregulated expression of HMGB-1, NF-κB, TNF-α, and vascular endothelial growth factor (VEGF) in T2D retinas and in cells treated with high glucose concentration. HMGB-1 blockade significantly attenuated NF-κB activity and VEGF secretion in these cells.^31^ Further studies by Chen et al (2013), indicated that HMGB-1 was significantly upregulated by high blood glucose concentration via NF-κB signalling, associated with increased expression of proinflammatory cytokines.^32^ HMGB-1 inhibition reduced the upregulation of proinflammatory cytokines in response to high blood glucose concentration. Therefore, HMGB-1 may play a role in the development of insulin resistance by activating NF-κB signalling and contributing to the elevated expression of proinflammatory mediators (refer Figure 2).^28,32^ HMGB-1 is believed to have a significant impact on insulin resistance by influencing NF-κB signalling.^28^ Following these reports, Chen and colleagues (2013), discovered that HMGB-1 expression, along with NF-κB and TNF-α/VEGF concentrations, were significantly elevated in the retinas of T2D patients and in ARPE-19 cells treated with high glucose concentration.^33^ Furthermore, blocking HMGB-1 resulted in reduced NF-κB activity and VEGF secretion in high glucose-stimulated ARPE-19 cells.^28^

Moreover, another study by Chen and colleagues (2015), demonstrated that high glucose upregulated HMGB-1 expression via NF-κB signalling both in *vivo* and in *vitro*, leading to increased levels of proinflammatory cytokines.^34^ Inhibition of HMGB-1 reduced the high glucose-induced upregulation of proinflammatory cytokines. Therefore, these findings suggest that HMGB-1 may contribute to the development of insulin resistance by activating NF-κB signalling and promoting the expression of proinflammatory mediators (refer Figure 2).^28,35^

## Cardiovascular complications: Implications of HMGB-1

**Figure 3.**
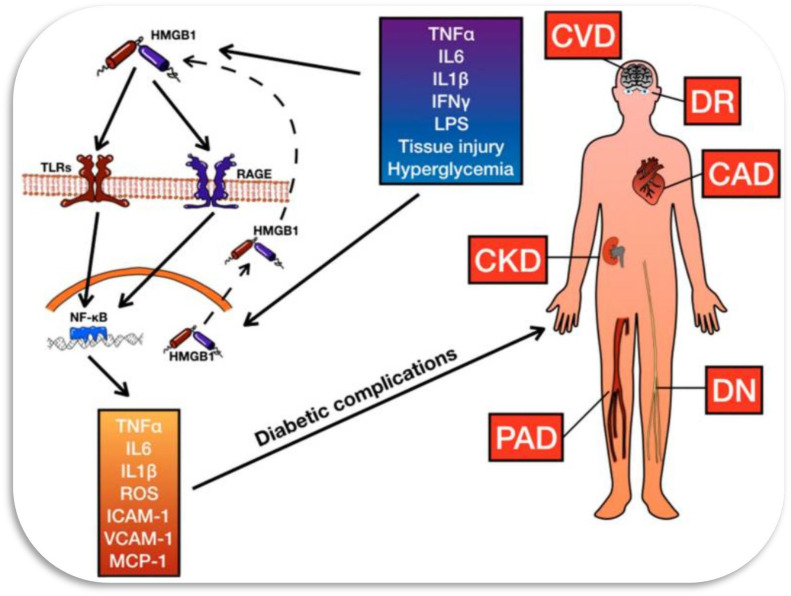
The HMGB-1 system influences various complications of diabetes, including coronary artery disease (CAD), cerebrovascular disease (CVD), diabetic retinopathy (DR), chronic kidney disease (CKD), diabetic neuropathy (DN), and peripheral artery disease (PAD). HMGB-1 affects these conditions by interacting with Toll-Like Receptors (TLRs), the Receptor for Advanced Glycation End Products (RAGE), and other pathways, leading to the production of inflammatory cytokines such as interleukin 1β (IL1β), interleukin 6 (IL6), and Tumor Necrosis Factor-α (TNFα). Additionally, HMGB-1 activates nuclear factor kappa-light-chain-enhancer of activated B cells (NFkB) and promotes the generation of reactive oxygen species (ROS), further exacerbating inflammation. This inflammatory response contributes to the pathogenesis of various diabetic complications by promoting endothelial dysfunction, increasing the expression of adhesion molecules such as Intercellular Adhesion Molecule 1 (ICAM-1) and Vascular Cell Adhesion Molecule 1 (VCAM-1), inducing the secretion of Monocyte Chemoattractant Protein 1 (MCP-1), and enhancing the activity of Interferon γ (IFNγ) and Lipopolysaccharide (LPS).

Cardiovascular complications associated with diabetes mellitus include atherosclerosis, hypertension, cerebrovascular disease, and coronary heart disease where arteries and veins are affected (refer Figure 3).^15,36^

## Vascular effects

The central pathological mechanism in cardiovascular disease is the process of atherosclerosis which leads to narrowing of arteries hence increased blood pressure.^2^ The risk of atherosclerosis is increased by the elevated circulating triglycerides and low-density lipoprotein (LDL) and decreased high density lipoprotein (HDL).^37^ Hypertension in diabetes is attributed to both haemodynamic and metabolic disturbances.^38,39^ Glucose homeostatic disturbances have been reported to cause endothelium dysfunction through various mechanisms. The endothelial dysfunction is associated with decreased nitric oxide (NO) and prostacyclin concentration and increased endothelin-1 concentration.^40^ Endothelin-1 is a well-known vasoconstrictor which also increases the expression of adhesion molecules such as intercellular adhesion molecules (ICAM) and vascular adhesion molecules (VCAM) which are implicated in cell to cell interaction, resulting in arterial stiffening (refer Figure 3).^15,^^41^ Chronic hypertension has been shown to be associated with diastolic dysfunction, cardiac muscle hypertrophy and cardiomyopathy due to increased cardiac workload.^42^ There are various markers which have been shown to predict the risk of cardiomyopathy. Pro-inflammatory cytokines such as TNF-α and the interleukin 6 (IL-6) family including cardiotropin-1 and C reactive protein (CRP) are highly expressed in cardiac failure.^43^ Similar to other macrovascular complications of diabetes, the development of diabetic cerebrovascular disease (CVD) is associated with various mechanisms including vascular endothelial dysfunction, increased arterial stiffness, and systemic inflammation. These elements contribute both to the atherosclerosis of cerebral vessels and to worse outcomes following an acute event.^44,45^

HMGB-1 has been implicated in the pathogenesis and progression of cardiovascular complications. HMGB-1 can be released passively from damaged pancreatic β cells or actively secreted by dendritic cells (DCs) and macrophages that infiltrate the islets. HMGB-1 is reported to be involved in the autoimmune response that leads to the destruction of pancreatic β cells in type 1 diabetes (T1D).^14^ It acts as a pro-inflammatory cytokine and a damage-associated molecular pattern (DAMP), contributing to the chronic inflammatory environment observed in T1D.^46^ In T1D, extracellular HMGB-1 exacerbates autoimmune progression by disrupting regulatory T cells.^47^ Individuals with T2D exhibit a low-grade systemic inflammatory condition, where HMGB-1, known as a late-stage inflammation mediator, plays a significant role in the development of T2D.^48^ A study by Zhang et al (2014), illustrated that metformin protected against hyperglycaemia-induced cardiomyocyte injury by suppressing the expression of RAGE and HMGB-1.^49^

Currently, there is limited evidence on the role of HMGB-1 in diabetic CVD (refer Figure 3). Nonetheless, similar to other diabetic complications, HMGB-1 might play a crucial role in diabetic stroke.^50,51^ In streptozotocin (STZ) induced diabetic rats, middle cerebral artery occlusion led to elevated serum levels of HMGB-1 and matrix metallopeptidase-9 (MMP-9), and increased expression of MMP-9, RAGE, and TLR-4 in brain tissue, especially in microglia was observed.^51^ A study by Ye and colleagues (2011) discussed treatment with niaspan, a slow-release form of niacin, reduced levels of HMGB-1, MMP-9, RAGE, and TLR-4.^51^ Niacin has a protective effect after cerebrovascular injury, reducing inflammation and enhancing endothelial function. It also increases levels of angiopoietin-1, which is involved in neuronal differentiation, vascular remodelling, and endothelial cell survival.^51,52^ Thus, niaspan could be a potential treatment to improve neurological outcomes after stroke.

Additionally, a study by Hu and colleagues (2016) found that intravenous injection of bone marrow stromal cells into T2D rats with experimentally induced middle artery occlusion reduced HMGB-1 and RAGE expression in the ischemic brain, improved functional recovery, and decreased blood–brain barrier leakage by increasing desmin and zonula occludens-1 (ZO-1) levels.^50^ These findings contrast with a previous study on T1D rats with middle artery occlusion, where Chen and colleagues (2011) observed that bone marrow stromal cell treatment decreased survival rates, increased blood–brain barrier leakage, brain haemorrhage, and vascular density, and promoted atherosclerosis by increasing intima thickness and collagen production in the internal carotid artery.^53^ Due to these conflicting results, further research is needed to clarify the role of bone marrow stromal cells in diabetic stroke as a potential therapeutic strategy for diabetic CVD.

## Myocardial effects

Current research suggests a close association between diabetic myocardial ischemia/reperfusion injury, oxidative stress, reactive oxygen species (ROS) elevation, and mitochondrial dysfunction.^54^ Oxidative-reductive reactions can trigger HMGB-1 translocation, leading to sustained activation of proinflammatory pathways and exacerbating myocardial injury via binding with RAGE.^55^ Some experts consider diabetic heart disease as primarily a mitochondrial disorder. HMGB-1 plays a crucial role in regulating mitochondrial autophagy.^49^ HMGB-1 also modulates heat shock protein beta 1 (HSPB1) to maintain mitochondrial morphology and control mitochondrial autophagy, thus contributing to myocardial remodelling following diabetic ischemia/reperfusion.^55^ However, excessive autophagy during ischemia/reperfusion in diabetic cardiomyopathy can worsen myocardial injury.^56^ Studies indicate that reducing HMGB-1 expression in diabetic rats can decrease infarct volume, enhance haemodynamics, and alleviate inflammation. Inhibition of HMGB-1 can mitigate myocardial ischemia/reperfusion injury by suppressing autophagy.^57^ Wu and colleagues (2017), demonstrated that HMGB-1 promotes myocardial ischemia/reperfusion injury in diabetic mice through mediation of mitochondrial autophagy.^54^

In both in vitro and in vivo settings, hyperglycaemia induces HMGB-1 ribonucleic acid (RNA) expression and increases HMGB-1 protein levels in myocardial cells and fibroblasts.^58^ Volz and colleagues (2010), reported diabetic mice with post-myocardial infarction remodelling exhibit elevated HMGB-1 levels, leading to enhanced inflammation and fibrosis. Knockdown of HMGB-1 and RAGE genes in these mice reduces inflammation and infarct size.^55^ Additionally, mice lacking RAGE show decreased HMGB-1 expression and reduced NF-κB activity in the heart, indicating that HMGB-1 action in myocardial cells is primarily mediated by RAGE and NF-Kb.^55^

In diabetic mice, HMGB-1 contributes to cardiomyocyte apoptosis through activation of the extracellular signal-regulated kinase 1 (ERK1) ERK/Ets-1 pathway, which regulates cell growth, proliferation, and apoptosis. Inhibition of HMGB-1 with HMGB-1siRNA reduces ERK and Ets-1 phosphorylation induced by hyperglycaemia.^59^ Furthermore, HMGB-1 is implicated in myocardial fibrosis.^29^ Wang and colleagues (2014), demonstrated that HMGB-1 increases TGF-β1 levels in cardiac fibroblasts, enhancing MMP activity, collagen I and collagen III expression. The inhibition of HMGB-1 reduces signalling through p38MAPK, ERK1/2, and JNK, crucial pathways in cardiac hypertrophy, fibrosis, and cytokine-mediated inflammation.^60^ Song and colleagues (2016), found cardiac expression of HMGB-1 in a hyperglycaemic environment is mediated by the PI3Kgamma/Akt pathway, and treatment with an antioxidant prevents PI3Kgamma/Akt signalling and HMGB-1 production, suggesting a potential therapeutic target for diabetic cardiomyopathy.^61^

Moreover, Zhang and colleagues (2010), demonstrated in diabetic mice that treatment with resveratrol induces a cardioprotective effect by reducing HMGB-1 expression and downregulating RAGE, TLR-4, and NF-κB signalling.^62^ Resveratrol also reduces oxidative stress, ameliorates myocardial fibrosis and inflammation, and decreases TNF-α and iNOS levels, highlighting its potential as a treatment strategy for cardiac dysfunction.^62^ Neutralizing antibodies against HMGB-1 effectively suppress HMGB-1 release from the heart, lower the LC3-II/I ratio, attenuate mitochondrial autophagy, and alleviate myocardial injury in diabetic mice.^63^ Therefore, anti-HMGB-1 therapy emerges as a promising approach to mitigate HMGB-1-induced inflammatory damage, suppress autophagy, and improve patient outcomes.^54^

## Diabetic nephropathy: implications of HMGB-1

Renal complications are amongst the leading causes of mortality and morbidity for both type 1 diabetes (T1D) and T2D and most patients experience the condition before they are diagnosed.^64^ Renal complications are precipitated directly or indirectly through four main molecular pathways which involve advance glycated end products (AGEs), the polyol pathway, protein kinase C (PKC) and hexosamine pathways.^65^ These pathways result in increased oxidative stress which ultimately influences and activates the renin angiotensin aldosterone system.^66^ The clinical hallmarks of renal complications include increased albumin excretion and a decrease in the glomerular filtration rate (GFR), both of which are associated with high blood pressure and electrolyte handling disturbances.^67^ Albuminuria doesn’t only predict renal disease but also serves as an independent cardiovascular risk factor. ^66,68^ These functional changes in the kidney occur as a consequence of morphological changes which include the thickening of the glomerular basement membrane, mesangial cell expansion and loss of podocyte, extracellular matrix deposition, glomerulosclerosis as well as tubular-interstitial fibrosis.^69–71^ These morphological changes are as a result of increased inflammation (TNF-α, IL-2) and expression of growth factors such as transforming growth factor (TGF) and vascular endothelial growth factor.^72^ Various studies have indicated elevated levels of HMGB-1 in diabetic nephropathy (DN), suggesting its involvement in the development of this condition. For instance, Kim and colleagues (2011), observed heightened expression of HMGB-1 in renal glomerular and tubular cells of diabetic rats, particularly in comparison to normal controls.^73^ Additionally, diabetic rats exhibited increased levels of RAGE and NF-κB, which are implicated in kidney injury by regulating various mediators such as TNF-α, IL-6, IL-1, ICAM-1, and GM-CSF (refer Figure 3).^15,73^

Other research has also shown elevated levels of TLRs, HMGB-1, and cytokines like TNF-α, IL-6, IL-1β, TGF-β1, ICAM-1, and MCP-1 in DN.^74–77^ Specifically, Lin and colleagues (2012), demonstrated increased expression of TLR-4 and HMGB-1 in renal tubules of individuals with diabetic nephropathy, with TLR-4 levels correlating with interstitial macrophage infiltration and glycosylated haemoglobin.^78^ Furthermore, hyperglycaemia induced TLR-4 expression via PKC, leading to elevated levels of IL-6 and CCL-2 through the IkB/NF-κB pathway. Knockdown of TLR gene in mice resulted in reduced IL-6 and CCL-2 levels, thus exerting a protective effect on the kidney.^78^ In contrast, Mudaliar and colleagues (2013), showed that high glucose concentrations exposure increased levels of both TLR-2 and HMGB-1, with HMGB-1 stimulating the NF-κB pathway. Silencing of TLR-2 interrupted NF-κB nuclear expression and HMGB-1-induced NF-κB-DNA binding.^74^ HMGB-1 is also believed to be implicated in diabetic nephropathy development through its modulation of autophagy.^79^ The Inhibition of HMGB-1 reduces apoptosis and injury in podocytes, delaying glomerular function deterioration caused by diabetes through the activation of Akt/mTOR signaling pathway and inhibition of autophagy.^80^

Interestingly, Zhang and colleagues (2017), demonstrated that glycyrrhizic acid (GL), an HMGB-1 inhibitor, reduced expression of HMGB-1, RAGE, TLR-4, and activation of ERK, p38 MAPK, and NF-κB pathways in kidney tissue of diabetic rats.^77^ GL also decreased serum and kidney levels of TNF-α, IL-6, IL-1β, MCP-1, ICAM-1, and TGF-β1.^77^ Moreover, a recent study by Jigheh et al. indicated that empaglifozin reduced renal levels of HMGB-1, RAGE, and TLR-4, thereby alleviating renal inflammation, suggesting a potential therapeutic approach for the disease.^81^

## Diabetic retinopathy: implications of HMGB-1

Diabetic retinopathy (DR) is the leading cause of visual disability and blindness in people with diabetes.^82^ Diabetic retinopathy is characterised by retinal vessel micro aneurysms, haemorrhages, and oedema.^2^ One of the primary changes in diabetic retinopathy involves loss of pericytes in retinal capillaries, which may lead to vascular failure and chronic hypoxia.^83^ Hypoxia is one of the major inducers of angiogenesis.^36^ Hypoxic conditions lead to the upregulation of hypoxia- inducible factor (HIF) and VEGF, which then promote the rapid formation of neovessels, resulting in exacerbated angiogenesis.^84^ The sudden establishment of angiogenic vessels leads to leakiness and malfunctioning of vascular system.^36^ Vitreous hemorrhage is also observed in DR, due to leaking of newly formed blood vessels, and jell-like substance fills the centre of the eye, thus impairing vision.^85^ Retinal detachment is also observed in DR, due to abnormal new blood vessels which promote scar tissue growth, the later pulls the retina away, ultimately causing spot floating vision.^86^ Moreover, leaking and new growth of blood vessels interrupt the fluid flow in the eyes and this causes the pressure to accumulate, in severe cases this damages the optic nerve which leads to permanent blindness.

HMGB-1 plays a crucial role in causing inflammation in the retina. It acts as a receptor for danger-associated protein patterns and can detect elevated glycemic levels as a stress signal evident in T1D and T2D.^87^ Patients with advanced diabetic retinopathy often show elevated levels of HMGB-1. HMGB-1 exerts its pro-inflammatory effects in retinal cells through binding to TLR-4^88^ and RAGE, as well as activation of ERK1/2 and NF-kB signaling pathways (refer Figure 3).^15,89^ Intravitreal administration of HMGB-1 enhances these pathways and downregulates TLR-2 and occludin expression, increasing retinal vaso-permeability.^89^ Additionally, HMGB-1 seems to inhibit insulin signaling in retinal cells via its link to RAGE and TLR-4.^88^

However, conflicting findings exist regarding HMGB-1’s direct role in retinal and choroidal neovascularization.^90,91^ While some studies suggest direct mediation of endothelial cells by HMGB-1, others propose that HMGB-1 induces pericyte death, responsible for vasopermeability and endothelial proliferation, through TLR-4-dependent production of reactive oxygen species and cytokines by glial cells.^90^ Notably, subretinal injection of HMGB-1 in rats did not induce neovascularization or modify expression of VEGF-A in glial cells.^91^

Furthermore, HMGB-1 promotes oxidative stress in the retina, inducing ROS-derived apoptosis in retinal cells. The administration of glycyrrhizic acid inhibits this effect. In diabetic rats, treatment with Polygonum cuspidatum extract i.e. a potent antioxidant known for its potential health benefits. Polygonum cuspidatum reduces HMGB-1, RAGE, and NF-κB expression, improving vascular retinal permeability and inhibiting tight junction leakage.^92^ Intravitreal injection of HMGB-1 siRNA in rats reduces retinal damage and cellular death, improving retinal function. In human retinal endothelial cells treated with high glucose, HMGB-1 siRNA reduces oxidative stress and cellular apoptosis. Additionally, exosomes derived from mesenchymal stem cells overexpressing miRNA-126 suppress HMGB-1 expression and NF-κB and NLRP3 inflammasome activity in human retinal endothelial cells.^93^ Lastly, protein kinase A (PKA) appears to inhibit cytoplasmic HMGB-1.^94^

Recent studies indicate an association between autophagy and the mechanisms underlying pathological neovascularization and neurodegeneration mediated by HMGB-1, thus influencing the development and progression of DR.^95^ The role of autophagy in DR is multifaceted. While autophagy may promote cell survival in the early stages of DR, excessive autophagy can lead to necrosis and exacerbate the condition. Intermittent hyperglycemic oxidative stress can modulate autophagy in retinal pigment epithelial cells, promoting cell survival by upregulating HMGB-1.^96^ HMGB-1 can translocate to the lysosome via the autophagy-lysosome pathway, leading to the release of lysosomal enzyme B into the cytoplasm, thereby inducing inflammation and apoptosis.^97^ Feng et al. demonstrated that HMGB-1 participates in lysosomal membrane penetration (LMP) and autophagy inhibition in retinal pigment epithelial (RPE) cells. Decreased HMGB-1 expression restored autophagic degradation, reduced inflammatory cytokine and VEGF expression, and protected RPE cells during the early stages of DR.^98^

## Diabetic neuropathy: Implications of HMGB-1

In clinical practice, diabetic neuropathy (DNE) is defined as signs and symptoms of peripheral nerve dysfunction in a diabetic patient where other causes of peripheral nerve dysfunction have been excluded.^64^ DNE has caused more hospitalization of diabetes mellitus patients than other complications, with half of the patients having some degree of the disease such as polyneuropathy and mononeuropathy.^99^ The prevalence of DNE has been found to be 66% and 59% for T1D and T2D, respectively.^100^ This complication is heterogeneous by symptoms and signs, risk factors, underlying mechanism and pathologic alterations. Mono and polyneuropathies, plexopathies and radiculopathies are observed in DNE.^101^ Autonomic neuropathy has been considered irreversible and life threatening since there is high risk of mortality. However, some studies have shown that cardiac denervation regresses with high glycaemic control.^102^ Pachydaki et al (2006)^103^ and Yu et al (2015), noted increased expression of HMGB-1 in the retinas of individuals with diabetes and rat models with retinopathy.^104^ As a proinflammatory mediator, HMGB-1 is involved in diabetic neuropathy by interacting with RAGE and TLR4 and regulating autophagy. Elevated HMGB-1 levels stimulate glutamate release and mediate neurotoxicity.^7^ Neuronal cells release HMGB-1 during seizures, accompanied by increased TLR4 expression.^105^ Guo and colleagues (2019), observed increased HMGB-1 and TLR4 protein expression levels and exacerbated neuronal apoptosis in KKAy mice subjected to intermittent hypoxia to induce diabetic neuropathy. Furthermore, HMGB-1 siRNA significantly reduced HMGB-1 and TLR4 protein expression, regulating autophagy, and reducing neuronal apoptosis.^106^ Several studies have explored the connection between HMGB-1 and DNE, building upon previous research highlighting HMGB-1’s involvement in the nervous system. For instance, HMGB-1 has been implicated in central ischemic damage, where it is released into the extracellular space following ischemic insult, promoting neuroinflammation (refer Figure 3).^15,107^ Inhibition of HMGB-1 expression has been shown to reduce infarct size and microglia activation.^107^ Additionally, in rat models, HMGB-1 promotes pain hypersensitivity after peripheral nerve injury, likely through RAGE activation, while treatment with anti-HMGB-1 antibodies alleviates hyperalgesia. Nerve injury also upregulates HMGB-1 mRNA expression in dorsal root ganglia (DRG) and spinal nerves.^108^

Furthermore, Zhao and colleagues (2016), demonstrated that calmodulin-dependent protein kinase IV (CaMKIV), a protein kinase involved in neuropathic pain, and HMGB-1 are upregulated in the dorsal root ganglia of rats treated with STZ, a substance used to induce diabetes and neuropathic pain.^109^ Inhibition of phosphorylated CaMKIV (pCaMKIV) decreases HMGB-1 levels and reduces thermal hyperalgesia and mechanical allodynia in diabetic rats, confirming the role of HMGB-1 in neuropathic pain.^110^

The expression of HMGB-1 in neuropathic pain also appears to be correlated with the Sigma-1 receptor (Sigma-1R), a receptor involved in nociception. STZ treatment induces expression of Sigma-1R and HMGB-1 in DRG, resulting in increased tactile allodynia and thermal hyperalgesia in rat models.^111^ Conversely, knockdown of Sigma-1R in rats shows modest tactile allodynia and thermal hyperalgesia in the absence of increased cytoplasmic levels of HMGB-1, suggesting a role of Sigma-1R in promoting HMGB-1 expression.^111^

Furthermore, the relationship between HMGB-1 and neuropathic changes induced by hyperglycaemia has been investigated in retinal neuropathy.^112,113^ Diabetes mellitus is associated with the activation of HMGB-1, activation of the ERK1/2 pathway, activation of cleaved caspase-3 (an apoptosis executer enzyme), and glutamate signalling pathways in rat retinas. Diabetic retinas also exhibit decreased levels of Glyoxalase-1 (GLO1), an enzyme important for detoxifying AGEs.

## Targeting HMGB-1: A promising approach

The understanding of HMGB-1 signalling and involvement in the onset and progression of diabetic complications could open further avenues for the prevention of diabetic complications. Despite tight glycaemic control afforded by antihyperglycaemics, diabetes complications inevitably develop, which underscore the necessity for extra-glycaemic protective agents. Aminoguanidine an AGEs formation inhibitor, failed in clinical trials primarily due to safety concerns and lack of efficacy. It’s therefore prudent to search for target that could yield similar protective effects. As we have highlighted above, HMGB-1 antagonism could protect against inflammation, oxidative stress, and AGEs. From a pharmacology perspective, HMGB-1 present yet another approach to prevent and delay the onset of diabetic complications which of often manifests despite glycaemic control. Metformin, a primary antidiabetic medication, is known to possess anti-inflammatory properties. Tsoyi et al (2011), showed that metformin significantly reduced HMGB-1 expression in LPS-treated RAW264.7 cells.^113^ Glycyrrhizin (GL) is a natural compound found in the liquorice plant, *Glycyrrhiza glabra*. It has been shown to directly bind directly to and inhibit HMGB-1, as reported by Mollica et al. (2007).^114^ GL and its derivatives exhibit significant anti-diabetic effects in diabetes mellitus and its associated complications.^115^ These effects include reducing blood glucose and insulin concentrations, improving insulin resistance and glucose tolerance, regulating lipid metabolism, and enhancing insulin secretion.^115^ However, GL’s bioavailability is limited, prompting the development of formulations such as nanoparticles or conjugation with various metals. GL-loaded nanoparticles have demonstrated efficacy in lowering blood glucose levels and improving lipid profiles.^115^ Notably, the dosages required when using nanoparticles are only one-quarter of those needed for pure GL, making nanoparticles a more potent option for GL delivery.^116^ Other HMGB-1 inhibitors include those that prevent the translocation of HMGB-1 from the cytoplasm and render its unavailability for extracellular proteins. Amongst these include ethyl pyruvate which has further been shown to inhibit RAGEs. Indeed, a recent study Jung et al., demonstrated that ethyl pyruvate prevents renal damage induced by methylglyoxal-derived advanced glycation end products.^116^ Wang et al recently demonstrated that ethyl pyruvate can reduce the inflammatory response after diabetic intracerebral haemorrhage and may inhibit the activation of inflammasomes by the HMGB-1/TLR4 pathway.^117^ Gabexate mesylate, a synthetic protease inhibitor is believed to inhibit plasminogen activator inhibitor-1 (PAI-1), and protease-activated receptor-2 (PAR-2), consequently, indirectly inhibiting HMGB-1 and mitigating tissue damage and/or diabetic complications.^118^ Other promising HMGB-1 inhibitors that could potential be considered for diabetes complications management include triptolide and diflunisal which have been shown to attenuate inflammation associated with HMGB-1.^119^ With a clear HMGB-1 understanding, we envisage it to be more appealing for drug discovery workers, to design and explore more potential HMGB-1 inhibitors.^120^

## Conclusion

HMGB-1, a chromosomal protein expressed widely and preserved throughout evolution, plays various roles in inflammation regulation. Mounting evidence suggests that HMGB-1 plays a crucial role in both the initiation and progression of diabetes. Elevated levels of HMGB-1 have been detected in the serum, islets, and other tissues such as adipose, liver, and muscle in individuals with diabetes and animal models. Moreover, diabetes is associated with increased expression levels of receptors like RAGE and TLRs, which play pivotal roles in triggering proinflammatory cytokines. The functional interplay between HMGB-1, RAGE, and TLRs exacerbates inflammation in T2D, including inflammation induced by obesity, insulin resistance, and islet inflammation. Thus, inhibiting HMGB-1 and its receptors emerges as a promising therapeutic strategy for managing inflammation in T2D.

## Data Availability

The authors confirm that the data supporting the findings of this study are available within the article.
